# The complete chloroplast genome of an endangered plant-*Nomocharis aperta*

**DOI:** 10.1080/23802359.2019.1696242

**Published:** 2019-12-09

**Authors:** Xueda Chen, Xiuhai Zhang, Yixin Liu, Junlian Gao, Zhen Xing, Yunpeng Du

**Affiliations:** a Resources and Environment College, Tibet Agriculture and Animal Husbandry University, Nyingchi, China;; b Beijing Key Laboratory of Agricultural Genetic Resources and Biotechnology, Beijing Functional Flower Engineering Technology Research Center, Beijing Agro-Biotechnology Research Center, Beijing Academy of Agriculture and Forestry Sciences, Beijing, China

**Keywords:** *Nomocharis aperta*, high-throughput sequence, complete chloroplast genome, phylogenetic analysis, *Lilium*-*Nomocharis* complex

## Abstract

*Nomocharis aperta* is an endangered and endemic species with high ornamental value in China. In this study, we reported a complete chloroplast genome of *N. aperta*, which was *de novo* assembled using the next-generation sequencing data. The complete chloroplast genome is 152,845 in length, including a large single copy region of 70,506 bp and a small single copy region of 17,468 bp and two inverted repeat regions of 26,520 bp. A total of 130 functional genes were encoded, consisting of 84 protein-coding genes, 36 transfer RNA genes, and 8 ribosomal RNA genes. The overall AT content of the chloroplast genome is 63.00%. In addition, phylogenetic analysis with the reported chloroplast genomes showed that *N. aperta* is nested within *Lilium* and close to *L. henricii*, *L. bakerianum* and *L. taliense*. It indicates that the study on the relationship between *Nomocharis* and *Lilium* needs more *Nomocharis* and *Lilium* complete chloroplast genome, especially some key species like *N. aperta*.

The genus *Nomocharis* was established by the famous French botanist Adrien Rene Franchet (1889) with *Nomocharis pardanthina* Franchet, based on material collected by Delavay in western Yunnan (Sealy 1983). However, it is still a problem about the classification of *Nomocharis* so far. Recent molecular phylogenetic analysis indicates that *Nomocharis* is nested within *Lilium* (Gao et al. 2012; Du, He, Wang, Wei, Li, et al. 2014; Du, He, Wang, Li, Wei, Yuan, et al. 2014). But several phylogenetic studies reported just use nuclear and chloroplast sequences, not complete genome. Now, we report the complete chloroplast genome of *Nomocharis aperta* in order to further study the classification of *Nomocharis* and solve the *Lilium-Nomocharis* complex deeply and completely.

*Nomocharis aperta* is an endangered species of *Nomocharis* (http://www.iplant.cn/rep/prot/Nomocharis%20aperta), samples of *N. aperta* were collected from Shangri-La (Geospatial coordinates: N：27°36′54″E：99°42′44″) in Northwest Yunnan, China, and DNA was stored at the herbarium of Institute of Botany, CAS (Herbarium number: BOP127294). Total genomic DNA was extracted from fresh leaves, according to the DNAsecure Plant Kit (Aidlab). A genomic DNA library was constructed using VAHTSTM Turbo DNA Library Prep Kit for IlluminaVR (Vazyme, Nanjing City, China). High-throughput sequencing was performed with pair-end reads on the HiSeq4000 Sequencing System at Novogene (http://www.novogene.com/index.php). The raw reads were quality-trimmed by NGSQC Toolkit v2.3.3 and assembled by SPAdes v3.6.1 (Bankevich et al. 2012). Assembled chloroplast genome was annotated using Dual Organellar GenoMe Annotator (http://dogma.ccbb.utexas.edu/) (Wyman et al. 2004). The gene map of the chloroplast genome was drawn in OGDraw v1.2 (Lohse et al. 2013).

The complete chloroplast genome of *N. aperta* (Genbank accession number: MN509269) is 152,845 bp in length, including a large single copy (LSC) region of 70,506 bp and a small single copy (SSC) region of 17,468 bp and two inverted repeat (IR) regions of 26,520 bp. The complete cp-DNA encodes 130 genes, comprising 84 protein-coding genes, 36 transfer RNA genes, and 8 ribosomal RNA genes. Among these genes, 15 genes (*trnK-UUU, rps16, atpF, rpoC1, trnL-UAA, trnV-UAC, rps12, petB, petD, rpl16, rpl2, ndhB, trnL-GAU, ndhA, trnA-UGC*) contained one intron, 2 genes (*ycf3, clpP*) contained two introns and 6 genes (*trnL-GAU, trnA-UGC, ndhB, rpl2, rps12, trnL-GAU*) were located in IR region. The nucleotide composition of *N. aperta* has high A + T content of 63.00%, and the corresponding values of the SSC, LSC, and IR regions were 69.40%, 65.30%, and 57.50%, respectively.

In order to analyze phylogenetic relationship between *N. aperta* and its related species, a maximum-likelihood (ML) phylogenetic tree was constructed with CIPRES (http://www.phylo.org/) (Miller et al. 2010). The complete chloroplast genome of 16 representative species from genera *Nomocharis*, *Lilium*, *Fritillaria*, *Notholirion*, and *Cardiocrinum* (later three genera as outgroups) were selected to perform the ML analysis. In the ML tree, *Fritillaria*, *Notholirion*, and *Cardiocrinum* were three separate monophyletic groups with the bootstrap values 100% (Figure 1). *Nomocharis aperta* was close to *N. pardanthina* (Liu et al. 2018) but nested within *Lilium*. It indicates that the phylogenetic position of *Nomocharis* needs a further study. So this report provides essential genetic data for further study on *Lilium-Nomocharis* complex. Meanwhile, it also contributes to conserve the genetic diversity of this endangered species.

**Figure 1.**
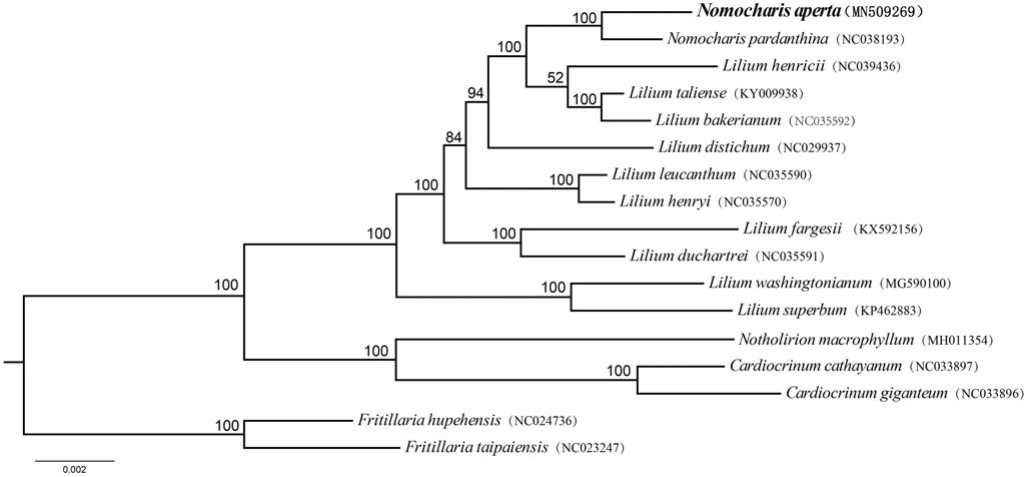
Phylogenetic relationships of 11 species in the *Nomocharis* and *Lilium* with the outgroups of 1 *Notholirion* species, 2 *Cardiocrinum* species and 2 *Fritillaria* species constructed by complets chloroplast genome with the maximum likelihood (ML) analyses. The bootstrap values were based on 1000 replicates.
